# Synchronous primary neoplasms of the bladder, skin and breast in a male patient: a case report

**DOI:** 10.1186/1477-7819-11-282

**Published:** 2013-10-20

**Authors:** Antonio Luigi Pastore, Giovanni Palleschi, Domenico Autieri, Antonino Leto, Andrea Ripoli, Cristina Maggioni, Davide Moschese, Yazan Al Salhi, Natale Porta, Claudio Di Cristofano, Andrea Fuschi, Luigi Silvestri, Carlo Della Rocca, Silverio Tomao, Vincenzo Petrozza, Antonio Carbone

**Affiliations:** 1 Department of Medical and Surgical Sciences and Biotechnologies, Unit of Urology, Faculty of Pharmacy and Medicine, Sapienza University of Rome, Corso della Repubblica 79, 04100, Latina, (LT), Italy; 2 Department of Medical-Surgical Sciences and Biotechnologies, Histopathology Unit-I.C.O.T, Latina, Italy; 3 Department of Medical-Surgical Sciences and Biotechnologies, Oncology Unit-I.C.O.T, Latina, Italy

## Abstract

The incidence of multiple primary malignant neoplasms increases with age, reflecting an increase in overall cancer risk in older patients. Cases of two or more concurrent primary cancers are still rare, although its incidence is increasing. Here, we report the case of a 57-year-old man who was referred to our institution with synchronous squamous cell carcinoma of the skin on the forehead, infiltrating ductal carcinoma of the breast, and transitional cell carcinoma of the urinary bladder. To the best of our knowledge, this is the first reported case in literature of this combination of primary neoplasms.

## Background

Multiple primary cancers (MPC) in a single patient was first documented by Billroth et al. in 1889 [1]. Since then, many cases of double, triple or even quintuple primary malignant neoplasms have been documented involving single or multiple organs [2]. MPCs are first classified as either synchronous or metachronous depending on their timing of diagnosis. Synchronous lesions are relatively uncommon, with most cases involving metachronous lesions [3,4].

However, there remains some confusion over the terms used to describe MPC, such as synchronous, simultaneous, and metachronous or successive neoplasms. All these definitions are based on the time that the neoplasms are discovered rather than the onset of disease. Thus, the term 'synchronous’ refers to neoplasms discovered simultaneously, while 'metachronous’ indicates a distinct neoplasm discovered when the same patient is already known to have a neoplasm (successive neoplasm) [5].

The generally accepted definition of MPC was introduced by Worren and Gates, who stated that each neoplasm must represent a distinct malignancy, and that a metastatic origin must be excluded [6]. Ray et al. reported that 13.5% patients with MPC had genitourinary neoplasms [7].

In this report, we describe the case of a patient who developed synchronous primary transitional cell carcinoma (TCC) of the urinary bladder, squamous cell carcinoma (SCC) of the skin on the forehead, and infiltrating ductal breast carcinoma. This combination, to the best of our knowledge, has never previously been reported in the literature.

## Case presentation

A 57-year-old man, a farmer and heavy smoker (90 to 100 cigarettes a day from the age of 15 years), was referred to our institute for gross haematuria with cloths retention that required an acute catheterisation with bladder irrigation. An ultrasound examination showed papillary neoplasms arising from the posterior-lateral left wall of the bladder.

The patient had suffered lower urinary tract symptoms over the preceding 1 year and seemed cachectic, but had not previously reported weight loss or any other specific complaints. He did have a family history of malignancy though, as his father had developed rectal cancer and his sister had a kidney neoplasm.

**Figure 1:**
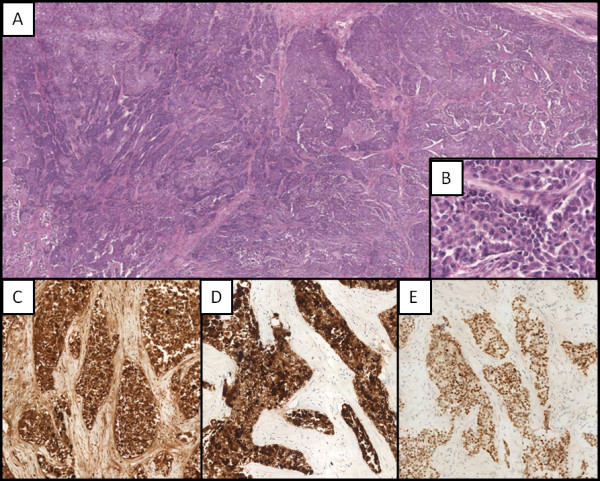
**Malignant breast neoplasia. (A)** Low-power photomicrograph breast epithelial proliferation mainly organized in solid nests and cords, with occasional pseudoglandular aspects (magnification 4x); **(B)** High-power photomicrograph showing epithelial cells of medium-great size, with altered nucleus-cytoplasm ratio, leptocromatinic nucleus, nucleolus often prominent and large eosinophilic cytoplasm (magnification 40x); the cell showed immunoreactivity for GCDFP-15 **(C)**, CK7 **(D)** and ER **(E)** (magnification 40x).

**Figure 2:**
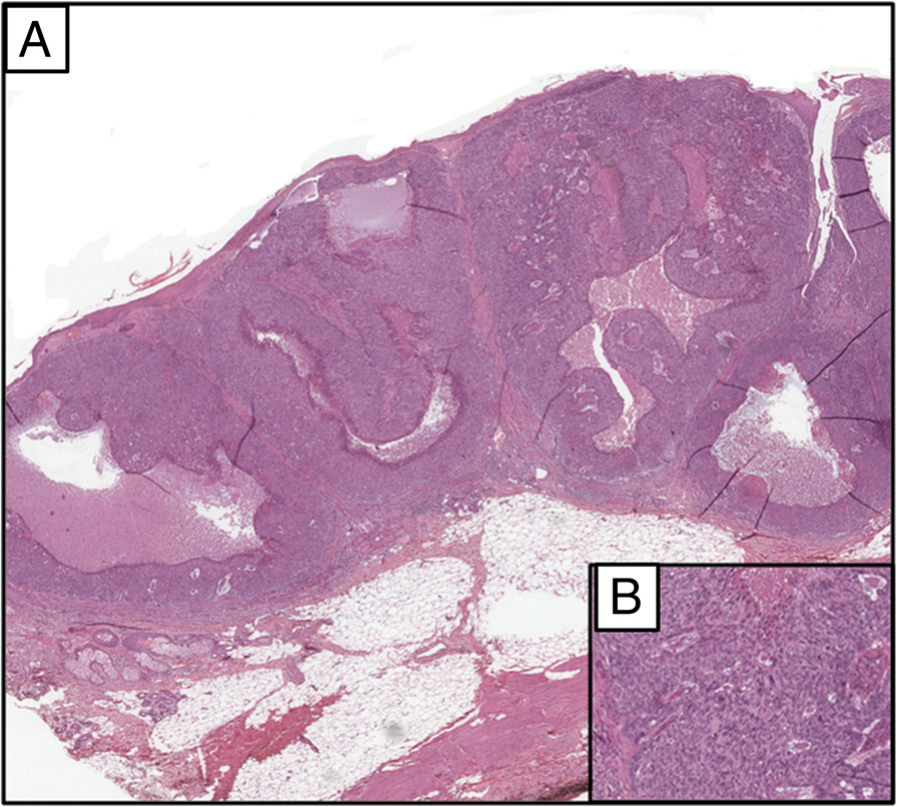
**Skin epithelial neoplasia. (A)** Low-power photomicrograph hypodermis infiltration, consisting of squamous cells arranged in solid nests and cords, with large areas of necrosis (magnification 4x); **(B)** High-power photomicrograph showing neoplastic cells characterized by moderately polymorphic nucleus, sometimes with evident nucleolus, large eosinophilic cytoplasm with ill-defined limits (magnification 40x).

**Figure 3:**
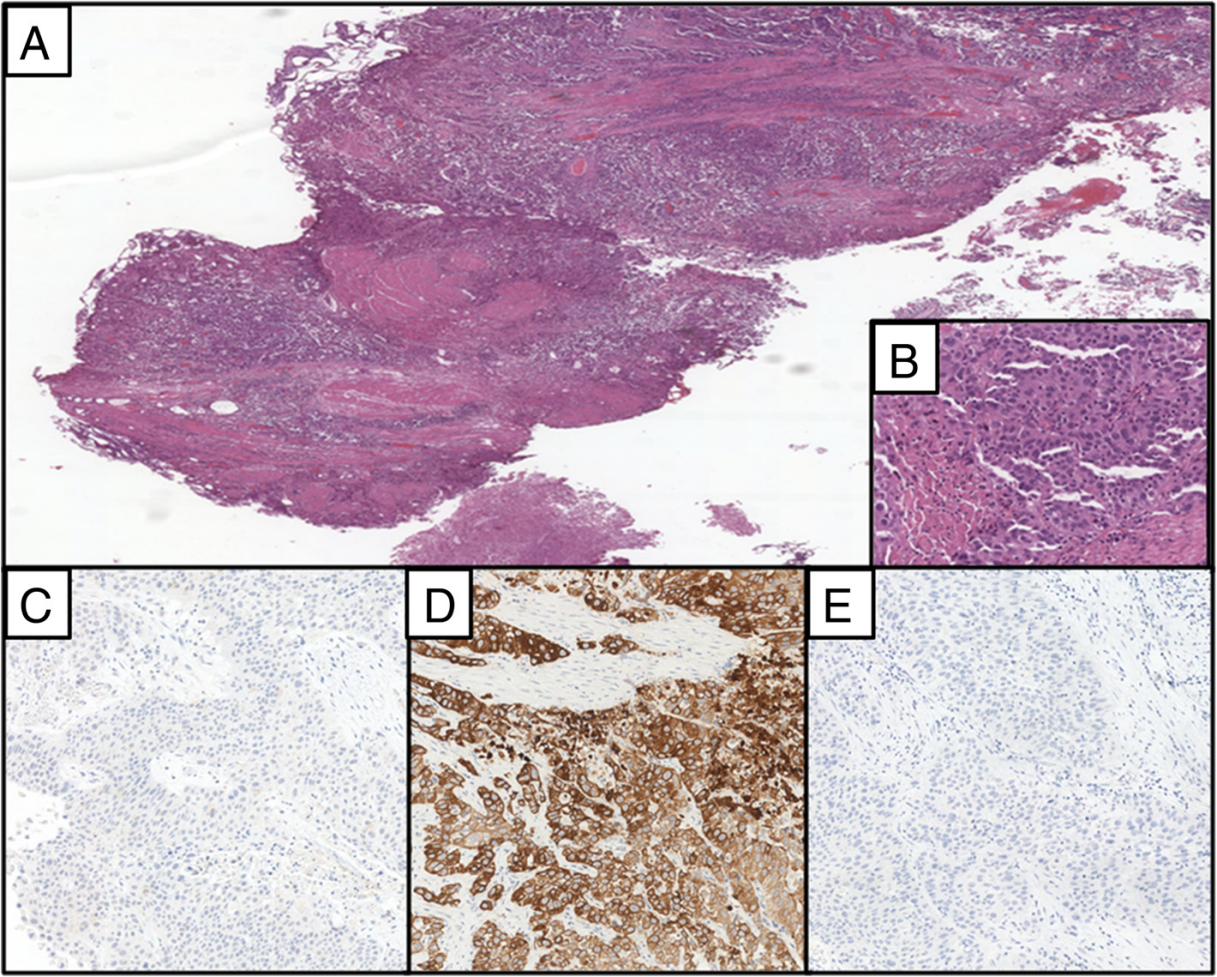
**Bladder cancer. (A)** Low-power photomicrograph showing malignant bladder epithelial proliferation organized in papillary structures, nests, sometimes with central necrosis, solid and cords that diffusely infiltrate subepithelial corium and the muscularis (magnification 4x); **(B)** High-power photomicrograph showing epithelial cells of medium-great size, with altered nucleus-cytoplasm ratio, leptocromatinic nucleus, nucleolus often prominent and large eosinophilic cytoplasm (magnification 40x); the cells resulted negative to immunohistochemical staining for GCDFP-15 **(C)**, positive for CK7 **(D)** and negative per CK20 **(E)** (magnification 40x).

He had no signs or symptoms until his physician noticed a hard lump with skin retraction on his left nipple. The patient also had an erythematous nodular skin lesion developing on his forehead. For these lesions, the patient underwent left modified radical mastectomy (based on the Madden technique) with axillary lymph node dissection. Histopathological examination revealed an infiltrating ductal carcinoma (grade III, score 8 according to Nottingham) with metastasis to one of the 11 axillary lymph nodes examined. Approximately 90% of the neoplastic cells stained positive with antibody to the oestrogen receptor, and 20% stained positive with antibody to the progesterone receptor. The proliferative index using a Ki-67 monoclonal antibody was 10%. HER-2/neu was not over-expressed (Figure 1). The nodular skin lesion was completely resected, and histopathological examination revealed it to be a SCC (grade II), infiltrating the hypodermis (Figure 2). Immediately after these surgical procedures, the patient was hospitalised in our institution where a cystoscopy was performed confirming ultrasound findings of multiple bladder papillary lesions arising from the posterior-lateral left wall, with a large base plant and active bleeding. A trans-urethral resection of the bladder lesion (TURB) was performed at the same time in which the neoplasm was completely excised. Subsequent histopathological examination showed a grade 3 papillary TCC that was in the process of infiltrating the muscular bladder wall (T2). The neoplastic cells were positive for cytokeratin 7 and negative for both cytokeratin 20 and Gross Cystic Disease Fluid Protein 15 (GCDFP-15) (Figure 3). Although a bone scan failed to find any skeletal metastasis, a whole body computed tomography (CT) scan revealed a diffuse thickening of the left bladder wall approximately 12.2 mm in diameter. This was found to be hypervascularised and in contact with the left vesicoureteral junction with minimal local infiltration of the perivesical area. There was also adenopathy in four retroperitoneal lymph nodes and concomitant left moderate hydroureteronephrosis. Therefore, the patient underwent total cystoprostatectomy with pelvic lymphadenectomy and a continent ileal urinary diversion. In consideration of the patient’s age and overall physical condition, an orthotopic bladder replacement (neobladder reconstruction) using the Paduan technique was chosen.

After surgery, the patient received adjuvant therapy (gemcitabine and cisplatin) for bladder and breast cancer, which were considered the most important prognostic factors among the three neoplasms. He is currently receiving adjuvant chemotherapy, including gemcitabine and cisplatin.

## Discussion

In case of synchronous MPCs, it is important to consider the stages of the different neoplasms, their likely biological behaviour, and the patient’s age, life expectancy, and co-morbidities as all of these can affect the treatment strategies and prognosis. The case we describe here met the criteria of Warren and Gates [6], namely that each neoplasm must be a distinct malignancy and not a metastasis of the other. In this patient, three primary and histologically distinct cancers were found in three different organs, that is, primary TCC of the urinary bladder, SCC of the skin on the forehead, and infiltrating ductal carcinoma of the breast, all within a 2-month period. All these cancers were considered primary neoplasms; thus, a diagnosis of synchronous triple primary cancer was made. Synchronous neoplasms are defined as ≥2 primary neoplasms diagnosed within 6 months of each other, while metachronous neoplasms are defined as those detected after an interval of >6 months [5]. A patient with a history of cancer may have an increased risk of developing another neoplasm due to exposure to common carcinogenic factors, such as tobacco and alcohol, or genetic predisposition (for example, Li-Fraumeni or Beckwith-Wiedemann syndrome), or as a side-effect of previous chemotherapy or radiotherapy [8]. In the current case, three likely causal factors were that the patient was a heavy, chronic smoker, that he had been exposed to chemicals, especially those in pesticides, and that he had experienced prolonged exposure to ultraviolet radiation from working outside. Gallagher et al. suggested that prior exposure to insecticides, herbicides, and fungicides or seed treatments are associated with an increased incidence of SCC of the skin [9]. No other predisposing factor or a family history was found that might have contributed to the development of these three neoplasms.

To our knowledge, this is the first reported case in the literature reporting this combination of primary neoplasms, although Pastore et al. previously described a case of synchronous ureteral and bladder metastases arising from infiltrating ductal breast carcinoma in an elderly woman [10]. We suggest that the onset of multiple primary neoplasms are the result of a combination of different factors, including improved cancer survival rates increasing the length of time over which additional cancers can develop [5].

## Conclusions

The incidence of MPC is probably influenced by a combination of environmental and genetic factors, and the prognosis for a patient with multiple malignancies is most probably determined by whichever neoplasm is the most aggressive. To the best of our knowledge, this is the first case in the literature to report distinct bladder, breast and skin primary neoplasms in the same patient.

## Consent

Written informed consent was obtained from the patient for publication of the case report and any accompanying images. A copy of written consent is available for review by the Editor-in-Chief of this journal.

## Abbreviations

CT: Computed tomography; MPC: Multiple primary cancers; SCC: Squamous cell carcinoma; TCC: Transitional cell carcinoma; TURB: Transurethral resection of bladder.

## Competing interests

The authors declare that they have no competing interests.

## Authors’ contributions

ALP, GP, AL, LS, AD, KS, AR, CM, NP, DM and AF were involved in the review of literature, acquisition of data and drafting and completing the manuscript. AC, CDR, VP and CDC conceived the study, participated in the co-ordination and the acquisition of data, and helped to draft the manuscript. All authors read and approved the final version of the manuscript.

## References

[B1] Billroth T [General surgical pathology and therapy*.* Guidance for students and physicians. Lecture] Khirurgiia (Mosk) 1991 10 10 136 143 1803082

[B2] Tiwari P Tripathi A Bansal P Vijay M Gupta A Kundu AK Synchronous primary cancers of urinary bladder and kidney and prostate Saudi J Kidney Dis Transpl 2012 23 786 789 10.4103/1319-2442.98161 22805392

[B3] Otrock ZK Mahfouz RA Salem ZM Four primary tumors of lung*,* bladder*,* prostate*,* and breast in a male patient South Med J 2005 98 946 949 16217994 10.1097/01.smj.0000173086.37625.e2

[B4] Komiyama S Nishio E Ichikawa R Miyamura H Kawamura K Komiyama M Nishio Y Udagawa Y Asymptomatic synchronous quintuple primary cancers Gynecol Obstet Invest 2012 74 324 328 10.1159/000339135 22776788

[B5] Koutsopoulos AV Dambaki KI Datseris G Giannikaki E Froudarakis M Stathopoulos E A novel combination of multiple primary carcinomas: Urinary bladder transitional cell carcinoma*,* prostate adenocarcinoma and small cell lung carcinoma*-*report of a case and review of the literature World J Surg Oncol 2005 3 51 10.1186/1477-7819-3-51 16045793 PMC1226150

[B6] Warren S Gates O Multiple primary malignant tumors. A survey of the literature and a statistical study Amer J Cancer 1932 16 1358 1414

[B7] Ray P Sharifi R Ortolano V Guinan P Involvement of the genitourinary system in multiple primary malignant neoplasms: a review J Clin Oncol 1983 1 574 6366144 10.1200/JCO.1983.1.9.574

[B8] Rabbani F Grimaldi G Russo P Multiple primary malignancies in renal cell carcinoma J Urol 1998 160 1255 1259 10.1016/S0022-5347(01)62510-2 9751330

[B9] Gallagher RP Bajdik CD Fincham S Chemical exposures*,* medical history*,* and risk of squamous and basal cell carcinoma of the skin Cancer Epidemiol Biomarkers Prev 1996 5 419 424 8781736

[B10] Pastore AL Palleschi G Tubaro A De Nunzio C Stoppaciaro A Silvestri L Serafini GM Stagnitti F Carbone A Synchronous urinary tract metastases from breast cancer Urologia 2009 76 66 67 21086298

